# Radiological impact of oral bisphosphonate use on polyostotic Paget's disease of bone over a 2 year period

**DOI:** 10.1016/j.radcr.2024.01.037

**Published:** 2024-02-23

**Authors:** Christopher Jude Pinto, Shadab B. Maldar, Siddhi Hegde, Sharanabasav M. Choukimath

**Affiliations:** a Department of Medicine, Karnataka Institute of Medical Sciences, Hubli, Karnataka, India; b Western Michigan University, Homer Stryker M.D School of Medicine, Battle Creek, MI, USA; c Department of Radiology, Massachusetts General Hospital, Boston, MA, USA; d Department of Pathology, Karnataka Institute of Medical Sciences, Hubli, Karnataka, India

**Keywords:** Paget's bone, Interventional radiology, Bone scintigraphy, Internal Medicine, fractures

## Abstract

Paget's disease of bone is a disorder of osteoclasts which hampers the physiological process of bone remodeling. It is the most common metabolic orthopedic disease in the Caucasian populace; we report the diagnosis of Paget's disease of bone in a South-Asian male in his early 50s with a history of gastrointestinal symptoms, weight loss and back pain. An alkaline phosphatase level of 1104 IU/L was noted. A 3-phase bone scan showed noncontiguous heterogenous nuclear uptake. After exhaustive evaluation, the patient was diagnosed with Paget's disease of bone. Despite the disease activity being mitigated by alendronate and monitored by ALP levels within normal range per protocol, the patient had compression fractures of the vertebrae requiring early reinitiation of oral bisphosphonates. This raised doubts about the efficacy of metabolic marker-based management in Paget's disease of bone.

## Background

Paget's disease of the bone (PDB) is a chronic benign disorder of the bone, characterized by well-defined osteolytic lesions in the long bones primarily affecting geriatric individuals [1]. It has an incidence of 1%-2% in the Caucasian population, although the exact incidence in the South-Asian populace is unknown, it is rare to be found in such a populace [2,3]. PDB is uncommon in people from South-Asia, especially in those under the age of 55 years. Consequently, cancer is a more probable etiology for a similar presentation in these settings [3]. Bone pain as a clinical symptom of PDB is likely to be seen in patients as they may experience warmth over the affected bone presumably due to increased vascular supply [1,2].

PDB is divided into lytic, mixed, and sclerotic phases. Findings at the lytic and mixed phases primarily involve raised alkaline phosphatase levels (ALP) and physiologic levels of calcium and phosphorus. In the PDB, bony tenderness, an increased incidence of pathological fractures, bone deformities and conductive hearing loss can be noted [1,2].

PDB has been noted to have an autosomal dominant pattern, with notable family history [1,2]. The progression of the disease could take months to years. PDB is usually detected incidentally in asymptomatic individuals who are found to have raised ALP levels or an abnormal radiograph while being investigated for other considerations. A total of 30%-40% patients can present with symptoms, most commonly bone pain. The diagnosis is made based on typical radiographic appearance [1], [2], [3], [4]. The extent of the disease is evaluated on the basis of radionuclide uptake scans. CT/MRI is indicated in patients with complications such as spinal stenosis or osteosarcoma [3]. Biochemical tests in PDB typically show an isolated elevation of ALP with otherwise normal results. Monitoring ALP levels help in diagnosis and assessment of treatment response, in otherwise asymptomatic patients [1], [2], [3], [4].

Current guidelines recommend the use of bisphosphonates and bone resorption markers to estimate bone turnover rates and radiological investigations in the settings of preventive care [1,2]. The guidelines aim to monitor the disease progress and improvement by graphing bone resorption markers with the aim to eventual baseline prepagetic levels [1], [2], [3], [4].

## Case presentation

A South-Asian male in his early 50s presented to our outpatient department, with complaints of dull aching back and pelvic pain for 8 months, with nocturnal accentuation, non-resolving on topical diclofenac usage. The pain was initially localized to the right side of his hip and then started affecting his lower back and then his upper back. The patient also complained of reflux symptoms resistant to pantoprazole for 6 months.

Past history showed a sprain with an avulsion fracture of the distal lateral malleolus of the left fibula 3 months ago. Alkaline phosphatase levels were 410 IU/L at the time of the fibular fracture. There was no other comparative history or lab work done prior. The patients did not have any primary care visits for the last 10 years.

Physical examination showed an averagely built male with stable vitals. The patient reported weight loss of 11 kgs (11.8% of body weight) in the last 6 months which the patient attributes to a diet change (reduced carb intake, high meat intake). Alcohol intake was limited to 1 glass of wine or 2 cans of beer per day. BMI was 27.8 kg/m^2^. On local examination, the neck was supple and showed no lymphadenopathy. Examination of the abdomen elicited mild epigastric tenderness. Examination of the spine elicited midline tenderness without any noted bony deformity.

## Investigations

Biochemical work showed raised alkaline phosphatase (ALP) levels of 1104 IU/L and mildly raised uric acid level of 10 mg/dL (Normal <6 mg/dL). Entire hemogram and metabolic panels for renal, lipid, and thyroid function were normal. Hepatic function testing in addition to ALP, normal. Prostatic specific antigen levels were 0.8 ng/mL (N < 3.5 ng/mL). Parathyroid Hormone levels were 24 pg/mL (N 10-60 pg/mL). His calcium levels were 9.4 mg/dL, a vitamin D level of 56 ng/mL and a phosphorus level of 3.9 mg/dL (within normal limits). Tumor marker Ca 19-9 and fecal occult blood testing were normal. Urine electrophoresis was normal.

Audiometric testing was normal. Radiological investigations with a whole body computed tomography (CT), showed no appreciable solid organ masses. However, during the CT, multiple lytic lesions were noted in the lumbar and thoracic vertebrae. With suspicion towards metastasis and supportive clinical findings of back pain, and weight loss, with raised alkaline phosphatase levels, a 99m-Tc uptake scan (3-phase bone scan) was performed. Full body skin imaging of moles with a dermoscope was normal. There were no cutaneous masses felt on palpation.

**Fig. 1:**
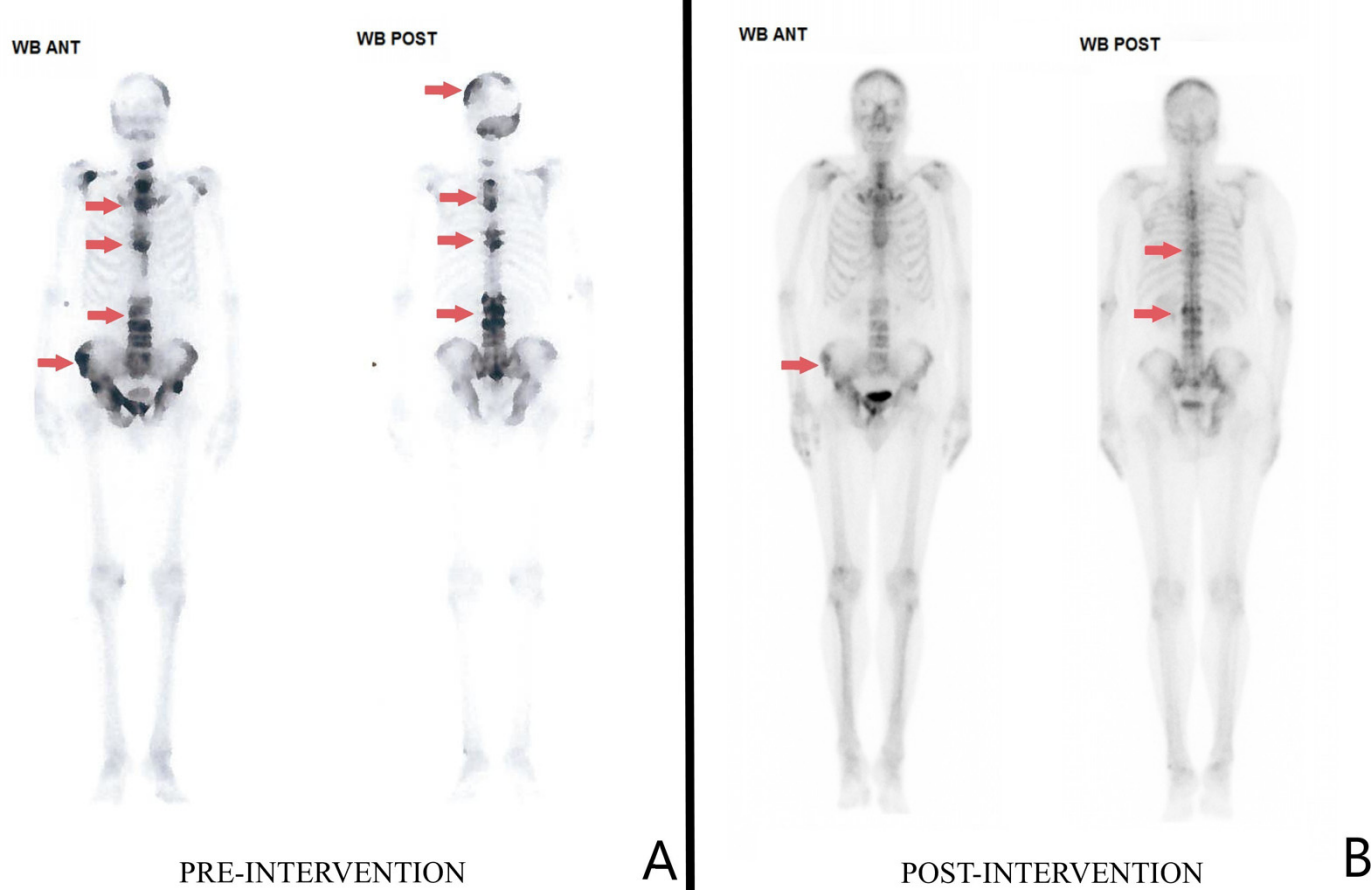
Comparative Tc-99MDP delayed phase scintigraphy bone scans showing heterogeneous uptake in anterior and posterior views. (A) Preintervention scan (prior to diagnosis). (B) Postintervention scan following 2 courses of 6-month oral bisphosphonate therapy. Areas of increased uptake noted in red across multiple appendicular sites and the skull.

Testosterone was within normal limits. Upper GI endoscopy and GI series were normal. MRCP and abdominal ultrasound findings were normal. The bone scan showed increased uptake in a non-contiguous heterogeneous pattern with multiple irregular foci (Fig. 1). Based on the uptake pattern, a provisional diagnosis of metastatic carcinoma with unknown primary was made with a differential of PDB, PDB related carcinoma and multiple endochondral cysts. Full body CT failed to show any notable abdominal, pulmonary, renal or cerebral masses.

**Fig. 2:**
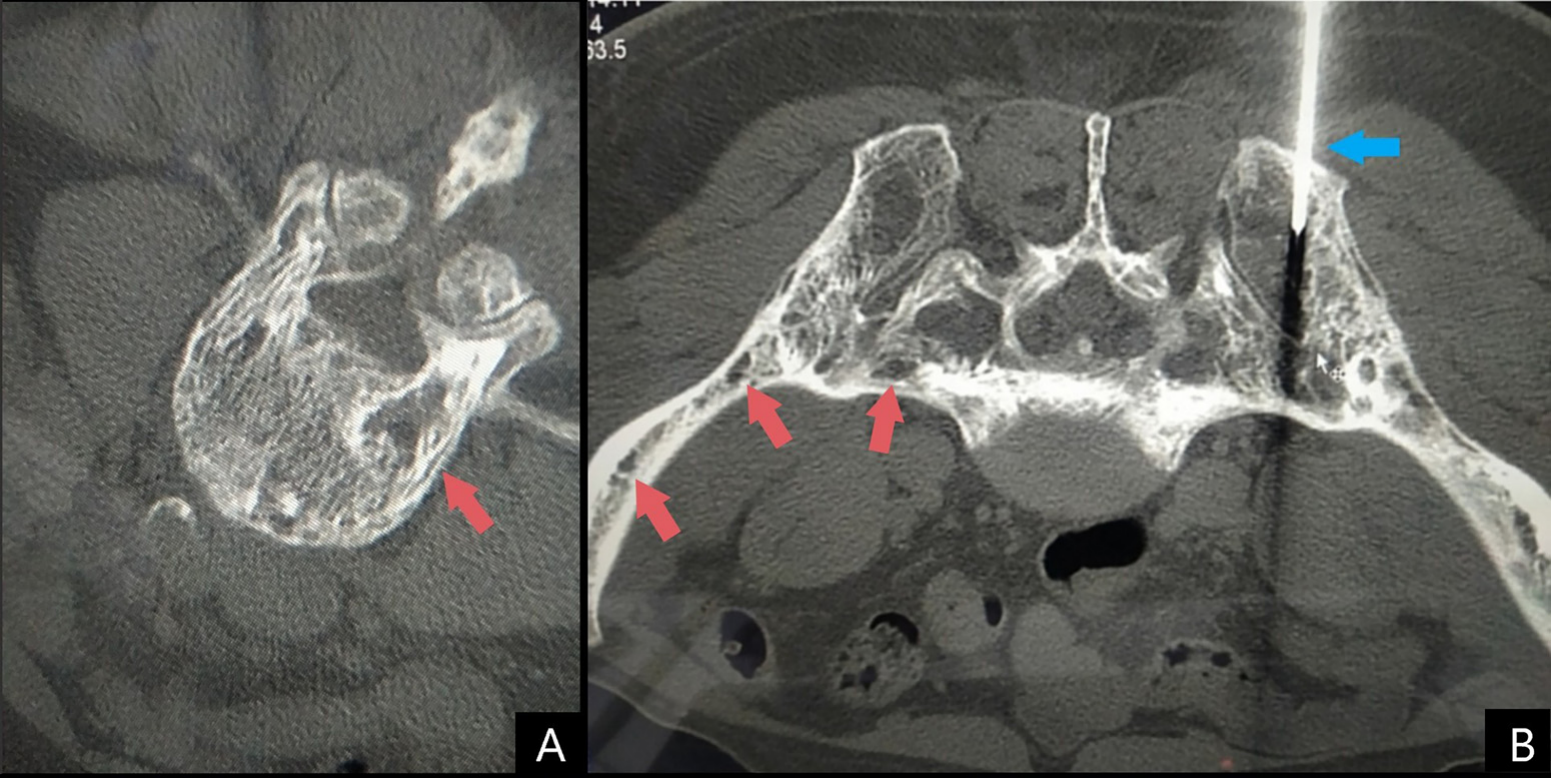
CT findings highlighting multiple lytic lesions of the spine and pelvis. (A) Lytic lesion on the L1 vertebra. (B) CT-guided bone biopsy (blue) of a lytic lesion in the pelvis. Multiple lytic lesions can be noted in the body of the pelvis and in the spine (red).

**Fig. 3:**
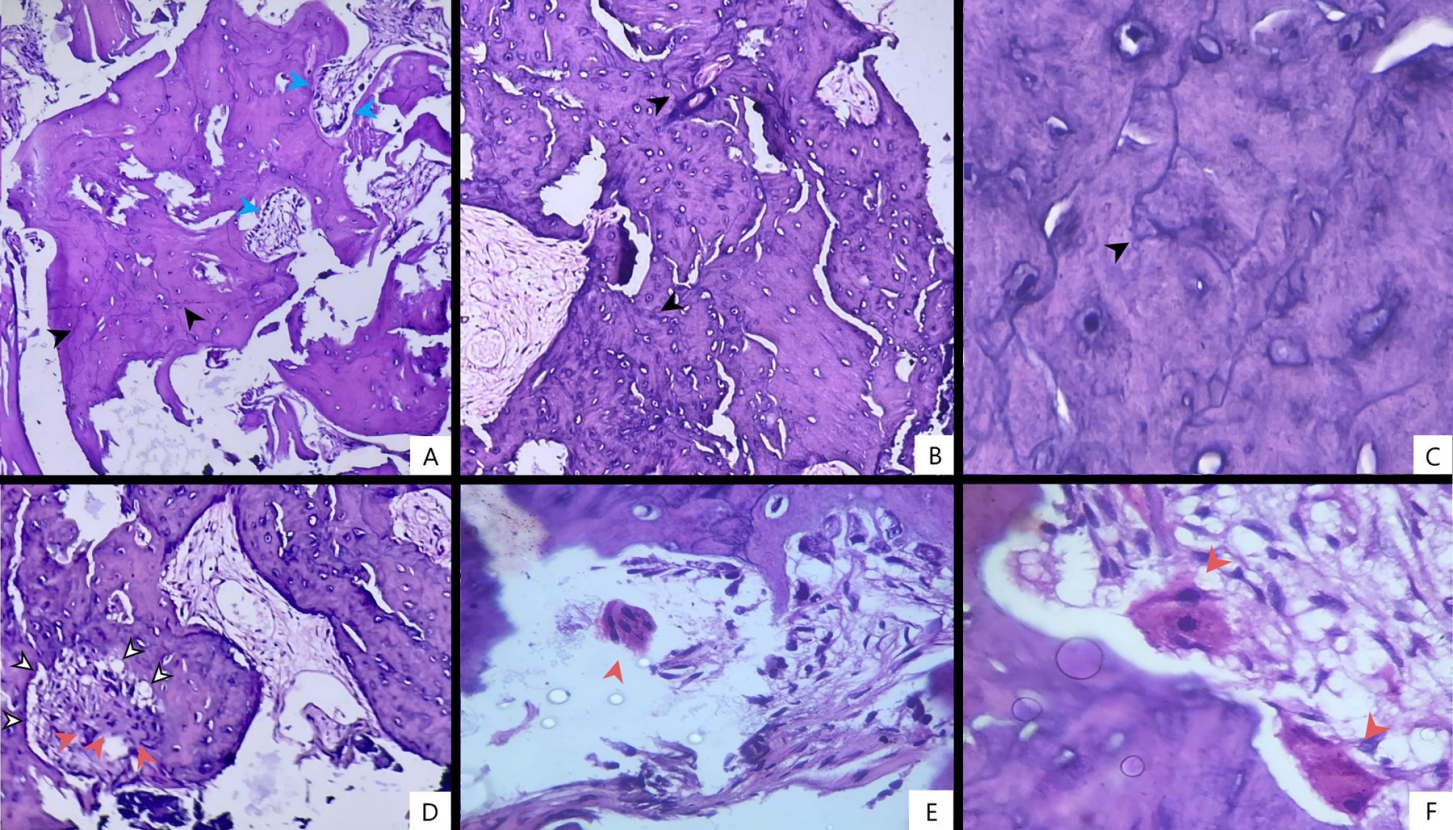
Histopathological specimens of the bone biopsy (H&E stain). (A,B,C) Showing characteristic mosaic patterns with cement lines with palisading osteoblasts, interspersed with woven and lamellar bone (10X,25X,100X). (D) Multiple giant osteoclasts with no dysplasia with areas of active resorption visualized– 25x (>8 nuclei per cell). (E) Multinucleated osteoclast (oil emulsion 100X). (F) Multiple multinucleated osteoclasts at bone divots indicating activity (oil emulsion 100X). Cement lines marked in black. Palisading osteoblasts marked in blue. Osteoclasts marked in red. Active resorption marked in white arrows with black outline.

Bone biopsy samples were acquired from the lumbar (L1 vertebra) and body of the pelvis (Fig. 2). The sections revealed no evidence of malignancy (Fig. 3). On immunohistology, bone biopsies were negative for CD117 and tryptase. Features were highly suggestive of Paget's disease of bone. Biochemical values, bone biopsy specimens and radiological features were confirmatory of polyostotic PDB in the lytic stage. Considering the presentation and progression of the disease, the patient's diagnosis of PDB was considered to be an atypical presentation due to the age group, and demographic.

## Treatment

The patient was started on oral bisphosphonates – alendronate 40 mg OD. The patient was advised to follow a sedentary lifestyle due to the risk of compression vertebral fractures and hip fractures. The patient was asked to follow up for an endocrinology evaluation and GI evaluation. IV zoledronate was considered as a first-line treatment, but due to the patients unvaccinated status at the crux of the COVID-19 pandemic, lack of available beds, admission for IV zoledronate administration was dissuaded.

## Outcome and follow-up

One month after initiation of treatment with oral bisphosphonates, repeat alkaline phosphatase levels were 633 IU/L, indicating a decrease of 43% from pre-intervention levels. Following alcohol cessation and change in heartburn medication to omeprazole 20 mg, the patient reported reduced exacerbations. The patient also reported a reduction in back pain with residual right hip pain.

**Fig. 4:**
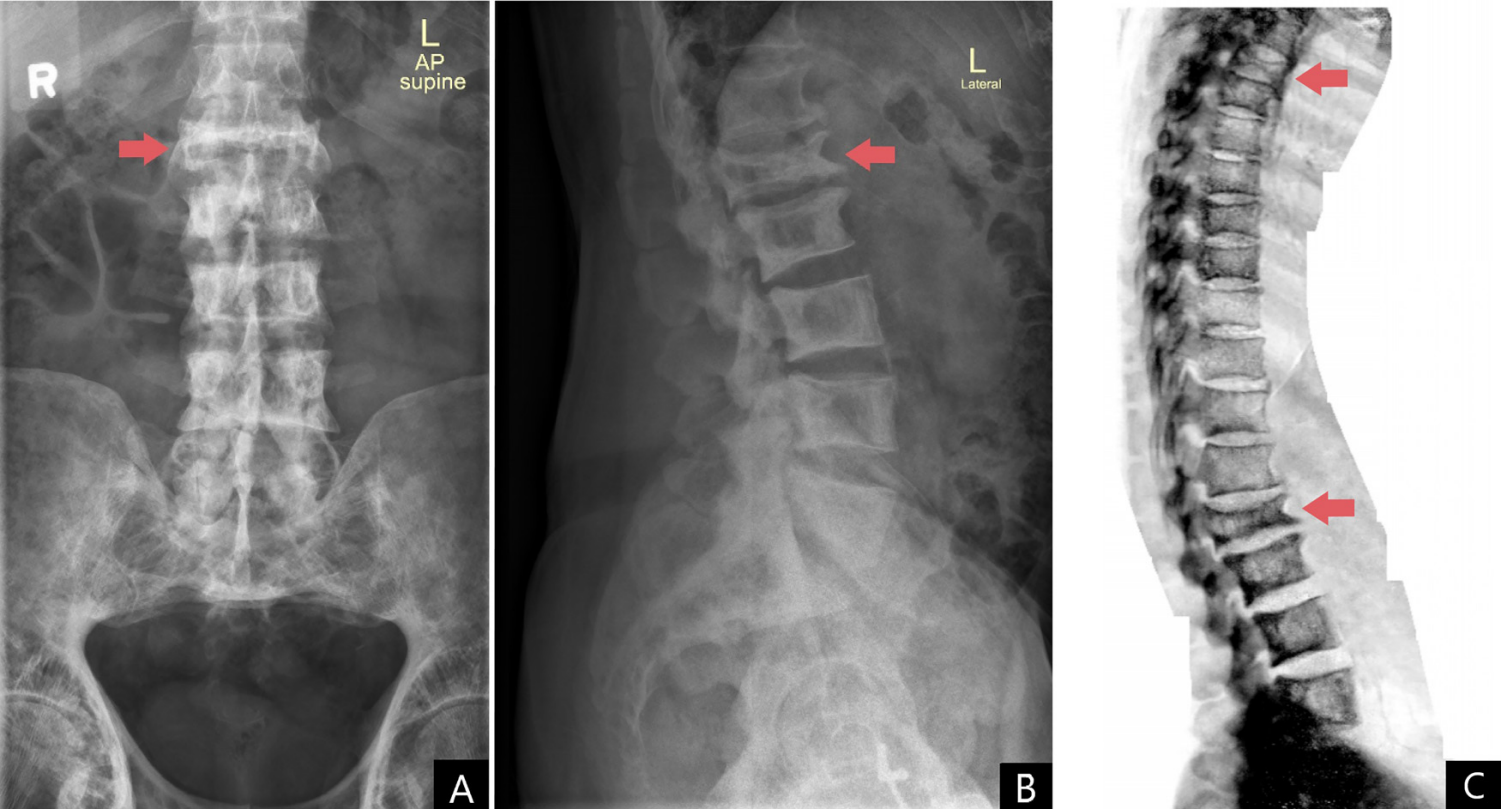
Follow-up x-rays and complications of compression fracture of T3 and L1 with lytic lesion in T12. (A) Lumbar AP view. (B) Lumbar lateral view. (C) Reconstructed full view spine. Compression fractures marked in red.

At the 6th month period, following completion of the course, the alkaline phosphatase levels were noted to be 58 IU/L, indicating appropriate response. Based on recommendations of the geriatrician, on a later visit due to persistence of mild right hip pain, bone specific ALP (b-ALP), urinary hydroxyproline (HP) and serum bone gla protein (BGP) were also done, which were within normal limits. 3 weeks later, (13 months from the time of diagnosis) the patient experienced sudden upper and lower back pain while getting up from his bed (low impact). The patient did not have any neural deficits. Plain radiographs were suggestive of compression fractures of the T3 and L1 vertebrae (Fig. 4). This corresponded with areas of increased uptake on the first bone scan as seen in Figure 1A. MRI of the spine showed no neural compression. The patient was restarted on oral bisphosphonates owing to previous response and for fracture healing and acceleration of trabecular bone formation. Calcium supplements (600 mg) with vitamin D3 (800 IU) were started on a daily basis.

**Fig. 5:**
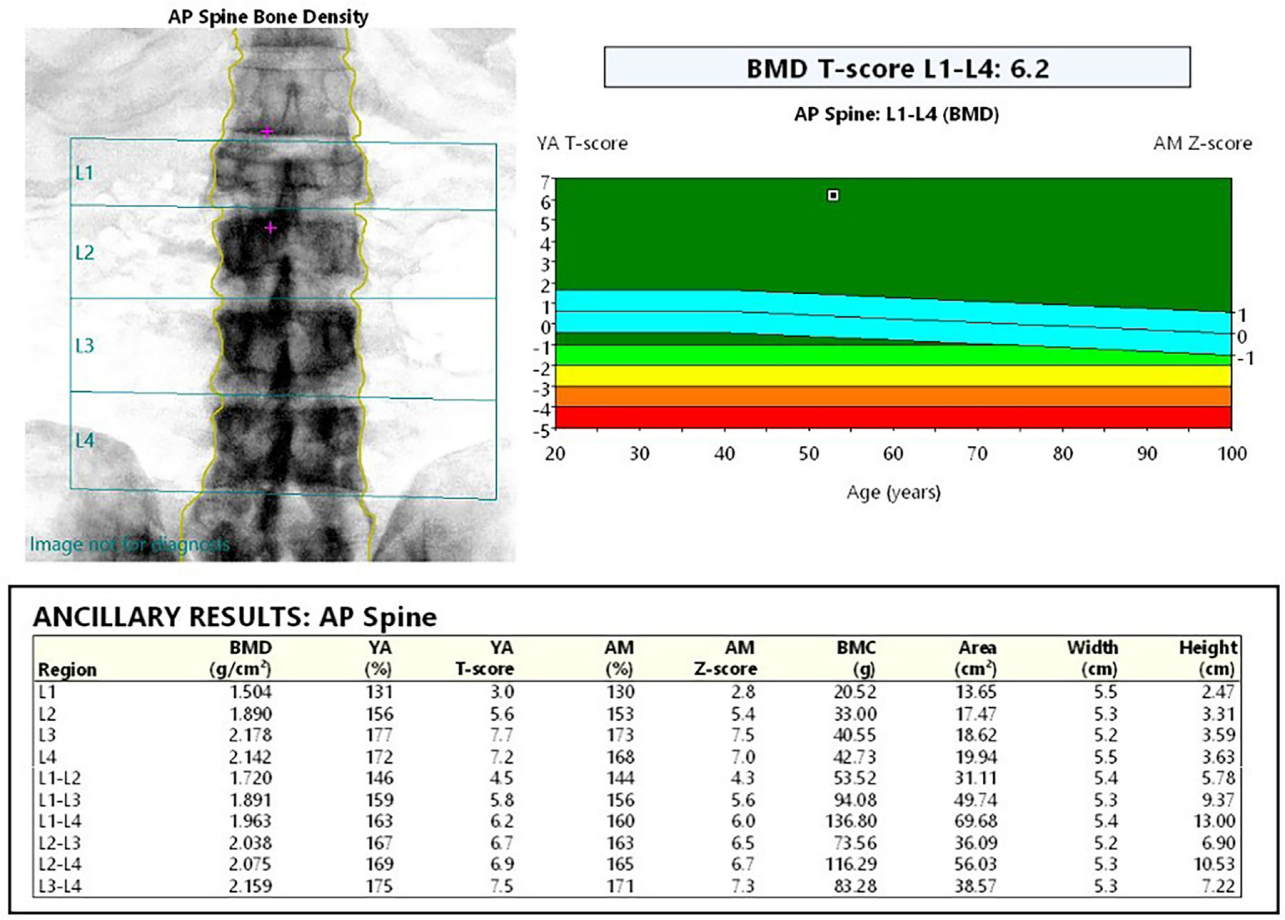
Dual x-ray absorptiometry of the lumbar spine following two 6-month courses of alendronate.

For evaluation of the disease process, a repeat 3-phase bone scan was done following completion of a second course of oral bisphosphonates (Fig. 1B). Findings of the 2nd 3-phase bone scan showed areas of diminished uptake as compared to the scan taken at the time of diagnosis. The alkaline phosphatase levels were 75 IU/L at the time of the uptake scan. A dual x-ray absorptiometry (DEXA) post 2nd course of oral bisphosphonates showed a raised lumbar bone mineral density (BMD) with a cumulative T score of 6.2, likely due to disease process and effect of bisphosphonate use (Fig. 5). The patient was given a back brace with restrictions to lifting weights heavier than 10kgs. The patient is currently being monitored every 3 months by his family physician.

The summary of events is present in Table 1.

**Table 1:** Summary of alkaline phosphatase records chronologically.

Timeline of events	Time frame from primary care visit	Notes/ALP levels (N < 150 IU/L)
Fibula fracture	0 d	ALP- 410 IU/L
Outpatient visit resulting in diagnosis of PDB/bone biopsy	3 mo	ALP - 1104 IU/L, Figures 1A, 2, 3
One month Rx bisphosphonates	4 mo	ALP- 633 IU/L
Six month RX bisphosphonates	6 mo	ALP- 58 IU/L
Monitoring visit	1 y and 1 mo	ALP - 124 IU/L, b-ALP- 19.5 μg/L BGP – 10.6 ng/mL, urinary hydroxyproline – 12.6 mg/24 hours/m^2^
Compression fracture of spine/Start of 2nd Rx Bisphosphonates	1 y 4 mo	Not recorded as ALP would be high due to fracture, Figure 4
Post 2nd Rx of bisphosphonates	2 y	ALP - 75 IU/L, Figures 1B, 5

**Legend 1**: ALP- Alkaline phosphatase, PDB- Paget's disease of bone, Rx - treatment.

## Discussion

Paget's disease of bone (PDB) occurs in 3 stages, the first being the lytic phase which involves rampant osteolysis in the appendicular skeleton and long bones. The osteoclastic dysfunction at this stage, leads to increased alkaline phosphatase levels (as seen in this patient, measuring 1104 IU/L) with normal levels of calcium and phosphorus. Mixed and sclerotic phases show remodeled bones with disorganized lamellar and woven structures leading to clinical presentations of bony deformities, fractures, and deafness. This disorganization decreases bone density and overall stability due to improper lattice formation, leading to the variable presentations of PDB. Fractures are noted in all phases of PDB [1], [2], [3], [4]. As seen in our case, the lack of fractures in the initial CT did not preclude to a state where they could be observed in later stages despite treatment. The development of future fractures herein was indicative of disease progression.

Heterogeneous technetium uptake and non-involvement of long bones made the diagnosis of PDB not as promising, as homogenous uptake and involvement of long bones are more commonly seen. Conditions such as multiple myeloma, malignant primary hyperparathyroidism, prostate cancer, systemic and bone-localized mastocytosis, osteoclast giant cell tumors, neuroendocrine tumors and cancers in general, often present with constitutional symptoms and specific features. Such differentials need to be ruled out with exhaustive investigations. Bony tenderness as a clinical feature seldom presents in the first phase, but in our case could have been an indicator of a florid course as indicated by the alkaline phosphatase levels of 1104 IU/L [1], [2], [3], [4], [5]. As per a systematic review of 332 patients with PDB, ALP levels studied were approximately 10 times higher than the normal limit with the mean average to be 1200.8 IU/L [6,7]. ALP values are found to be significantly higher in patients above the age of 40 [6,7]. Coincidental extrinsic exaggerating factors leading to elevated levels could include micronutrient deficiency and parathyroid causes, but these investigations yielded negative results in our patient. The extent of lesions and symptom profile indicate that the disease process likely evolved over years. Raised ALP levels at the during the fibula fracture were attributed to the fracture, and likely a missed opportunity for further investigations into etiologies such as PDB.

As per recommendations, monitoring of bone turnover markers such as ALP help in identifying metabolically active cases [1,2]. But in our case, the compression fractures despite normal bone turnover markers (b-ALP, urinary HP, BGP) were representative of an active disease process and likely progression into the mixed phase of PDB.

The significant diminished uptake in comparison to the prior bone scan showed a reduced uptake pattern following two 6-month courses by alendronate over a 2-year period. PDB can be medically treated with oral bisphosphonates to repair the improperly structured bone [1], [2], [3], [4]. The first line bisphosphonate treatment is with IV zoledronate with a 5mg infusion over 15 minutes; due to added costs and requiring admission during covid and due to lack of available beds, this option was not chosen as the patient was unvaccinated. Alendronate was covered by the institution and was started shortly after the bone biopsy. At the time of the vertebral compression fractures, the choice was offered to the patient again, and he chose alendronate due to no out of pocket costs. A potential confounder to the presentation is due to the patient's inability to undergo IV zoledronate treatment which is associated with better treatment outcomes in comparison to alendronate [1].

Bisphosphonates work by inhibiting osteoclastic action and show osteoclastic apoptosis. They selectively act on hydroxyapatite binding sites, preventing cells participating in active resorption – osteoclasts, to bind to these sites thereby preventing resorption [1], [2], [3], [4]. They are contraindicated in patients with previous recorded pill induced esophagitis, uncontrolled GERD and renal dysfunction. Though our patient had a history of heartburn (with no significant findings on upper GI scope), with complete cessation of alcohol and a changing over to another PPI enabled continuation of alendronate with close monitoring.

A key differential in this case was of malignant transformation of PDB from the polyostotic form, hence requiring the bone biopsy. Complications of PDB include <1% change of neoplastic transformation into osteosarcoma, chondrosarcoma and fibrosarcoma. Such changes are due the tumor-specific loss of heterozygosity zones on chromosome 18 [2,8]. Bone biopsies are usually not indicated in general presentations of polyostotic PDB with isolated raised alkaline phosphatase levels, but herein due to heterogenous uptake as seen in Figure 1A, they were performed. Biopsies are indicated in cases of clinical suspicion and of that in monostotic lesions due to association with other mimicking conditions as priorly mentioned [1], [2], [3], [4].

The recommendations provided by academic societies on PDB have footnoted that the pragmatic application of these guidelines may not ensure a successful outcome in every case [1,2]. Due to observable long term follow-up outcomes, the non-conclusive laboratory testing in our patient did not reflect the true disease process and response to medications, hence indicating the need for imaging in patients with such presentations and unclear responses. The ability of the physician to navigate through the disease process is based on clinical evidence and findings. Moreover, this case highlights the necessary judgment that healthcare professionals have to make in settings of diseases of the unconventional nature in such a demographic [2].

## Conclusion

Paget's disease of the bone (PDB) is a chronic benign disorder of the bone, characterized by well-defined osteolytic lesions in the long bones primarily affecting male individuals above 55. It has an incidence of 1%-2% in the Caucasian population, with the exact incidence in the South-Asian populace unknown, it is rare to be found in such a demographic. Although PDB is monitored by sequential alkaline phosphatase graphing with response to bisphosphonates, metabolic normality may not indicate an inactive disease process. Evident failure of laboratory tests to reflect true disease indicates a potential role to include imaging in the surveillance of these patients. The ability of the physician to navigate through the disease process is based on clinical evidence and findings, highlighting the necessary judgment that healthcare professionals have to make in settings of diseases in an atypical progressive course.

## Patient consent

Consent for publication was obtained directly from the patient. No physical image findings have been provided.

Consent was also obtained for the usage of the images in this paper directly from the patient.

The patient is still under follow-up.
